# Ophthalmic Parasitosis: A Review Article

**DOI:** 10.1155/2012/587402

**Published:** 2012-09-16

**Authors:** Amal R. Nimir, Ahmed Saliem, Ibrahim Abdel Aziz Ibrahim

**Affiliations:** ^1^Division of Basic Medical Sciences, Faculty of Medicine, Cyberjaya University College of Medical Sciences, 63000 Selangor, Malaysia; ^2^Department of Pharmacology, Faculty of Medicine, University Technology MARA, 40100 Shah Alam, Malaysia

## Abstract

Ocular parasitosis in human is more prevalent in geographical areas where environmental factors and poor sanitary conditions favor the parasitism between man and animals. Lesions in the eye can be due to damage directly caused by the infectious pathogen, indirect pathology caused by toxic products, or the immune response incited by infections or ectopic parasitism. The epidemiology of parasitic ocular diseases reflects the habitat of the causative parasites as well as the habits and health status of the patient. An ocular examination may provide clues to the underlying disease/infection, and an awareness of the possibilities of travel-related pathology may shed light on an ocular presentation. This paper is a comprehensive review of the parasitic diseases of the eye. The majority of the clinically important species of parasites involved in eye infection are reviewed in this paper. Parasites are discussed by the disease or infection they cause.

## 1. Introduction

Ocular parasitosis in human is more prevalent in geographical areas where environmental factors and poor sanitary conditions favor the parasitism between man and animals. In recent years, population shift and rapid transport have facilitated the spread of certain parasitic diseases from endemic to nonendemic areas. The routes of infection to man vary with species of the parasite and the animal hosts they infest. Lesions in the eye can be due to damage directly caused by the infectious pathogen, indirect pathology caused by toxic products, immune response incited by infections, or ectopic parasitism of the preadult or adult stages. 

The epidemiology of parasitic ocular diseases reflects the habitat of the causative parasites as well as the habits and health status of the patient. Additional consideration must include local sanitation and the presence of a vector for transmission as well as the more complicated life cycles of the parasites and definitive hosts. Dietary history should be considered since most parasitic transmission is through food and water contamination. Travel history to endemic areas is important to determine the source of infection. An awareness of these is therefore important to the clinician evaluating this group of patients. 

An ocular examination may provide clues to the underlying disease, and an awareness of the possibilities of travel-related pathology may shed light on an ocular presentation. The eye is involved both in a variety of systemic infections and may be the primary focus of other pathologies. The majorities of conditions affecting the eyes—other than injuries—are infectious.

In some occasions, the ophthalmic lesions occur as a result of antiparasitic treatment as it has been noticed in the prophylactic and therapeutic attempts to treat malaria [1, 2]. Drugs such as hydroxychloroquine and chloroquine can damage vision because of their toxic effects, which is due to slow accumulation of the drugs in the retinal epithelium that results in irreversible visual loss. Much debate and confusion have taken place over the type and frequency of ocular examination in patients taking these drugs. 

Despite improved understanding of the clinical features of inflammatory eye diseases and advances in diagnostic testing, clinicians should maintain a high index of suspicion for infective parasitic diseases in patients thought to have inflammatory eye involvement. 

Because of this somehow more complex scenario, and the tendency for the parasites to cause a wider variety of pathologic lesions, the various parasitic etiologies of ocular diseases will be addressed individually, including epidemiology, pathology, diagnosis, and treatment.

## 2. Protozoan Eye Infection

### 2.1. *Acanthamoeba Keratitis*

*Acanthamoeba* spp. is ubiquitous free-living protozoa that have been isolated from several habitats, including soil, bottled water, eyewash stations, and air. 

There are two stages in the life cycle of this environmental ameba: the motile trophozoite (8–40 *μ*m) and the dormant cyst (8–29 *μ*m) [3]. By encysting, *Acanthamoeba* spp. can evade extreme environmental conditions such as hyperosmolarity, glucose starvation, desiccation, extreme temperatures, and extreme pH [4].

The leading risk factors for *Acanthamoeba* keratitis are contact-lens wear and corneal trauma [5, 6]. Although >80% of the cases of *Acanthamoeba* keratitis occur in contact lens, in other countries such as India, keratitis that is not related to contact lenses commonly occurs after corneal trauma or exposure to contaminated water [7]. One clinical manifestation of *Acanthamoeba* keratitis is radial neuritis and severe pain that is not commensurate with the extent of tissue damage. It typically presents as a unilateral central or paracentral corneal infiltrate, often with a ring-shaped peripheral infiltrate. Other characteristic symptoms (which appear in the early phases of infection) include eyelid ptosis, conjunctival hyperemia, epithelial ulcers, and lack of discharge. These symptoms are often followed by the appearance of a ring-like stromal infiltrate in the later stages of disease. *Acanthamoeba* keratitis can progress to scleritis, and, in severe cases, uncontrolled infections require the removal of the affected eye [8]. Interestingly, the pathogenic cascade of *Acanthamoeba* keratitis has interesting parallels with the amebic colitis caused by *E. histolytica *[4].

A provisional diagnosis of AK can be made using the clinical features and confocal microscopy although a definitive diagnosis requires culture, histology, or identification of *Acanthamoeba* deoxyribonucleic acid by polymerase chain reaction [9]. Trophozoites and cysts can be identified in Giemsa or periodic-acid-schiff-stained smears from scrapings or corneal biopsy specimens. Culture of *Acanthamoeba* spp. requires growth on nutrient agar plate seeded with bacteria. Treatment of amebic keratitis is difficult and disappointing. Long-term topical application of agents such as propamidine, miconazole, and neomycin has been successful in only few instances. 

### 2.2. Chagas' Disease

Chagas' disease, or American trypanosomiasis, results from infection by *Trypanosoma cruzi*. Infection occurs when an infected reduviid triatomine bugs bites a human. Once introduced, the trypomastigotes circulate throughout the body with a preference for invading muscle cells, neural tissue, and the reticuloendothelial system. If the initial bite is near the orbit, the patient may experience significant palpebral and periorbital oedema (Romana's sign). The edema is usually painless and is frequently followed by constitutional symptoms of fever, malaise, and anorexia. 

The diagnosis of acute Chagas' disease is made by the detection of trypomastigotes in the bloodstream by direct examination of uncoagulated blood or buffy coat preparation. Direct culturing of blood on Novy, MacNeal, Nicolle's medium (NNN) or other suitable media may result in positive cultures in 7to 10days [10]. The technique of xenodiagnosis may be used for diagnosis, if available. Serologic testing is of little value in the diagnosis of acute Chagas' disease as antibodies do not usually appear for 2to 40days following the onset of symptoms. Additionally, serologic studies may falsely detect the cross-reactivity of antibodies to nonpathogenic *Trypanosoma rangeli* [11].

Therapy of Chagas' disease with antitrypanosome therapy is most successful in the acute stage. Two medications are available: nifurtimox and benznidazole. Therapy is usually extended for a period of months, and parasitologic cure rates are somewhat disappointing. Both medications carry a long list of significant side effects.

### 2.3. Giardiasis

The protozoan disease giardiasis can cause ocular complications, including “salt and pepper” retinal changes. One study showed that asymptomatic, nonprogressive retinal lesions are particularly common in younger children with giardiasis. This risk does not seem to be related to the severity of the infection, its duration, or the use of metronidazole but may reflect a genetic predisposition [12].

Diagnosis is confirmed by finding the cyst stage in the fecal smear. Treatment is the same as for intestinal infection, that is, metronidazole. 

### 2.4. Leishmaniasis

*Leishmania *spp. is obligate intracellular protozoans which infect an estimated 12million persons. There are numerous species within the genus, and disease manifestation is, in part, species specific. 

Once injected into humans during the sandfly blood meal, the promastigote develops into an amastigote after being engulfed by tissue macrophages. Within these cells, the amastigotes replicate and may spread either systemically or cutaneously. 

Visceral leishmaniasis, or that which represents systemic disease, is known as kala-azar. The ocular manifestations of kala-azar are relatively uncommon and include chorioretinitis, central retinal vein thrombosis, iritis, papillitis, and keratitis [13]. Additionally, flame-shaped retinal hemorrhages have been described. Glaucoma has been reported to develop after the successful treatment of kala-azar. 

Ocular findings in cutaneous leishmaniasis represent a local phenomenon resulting from the initial site of infection near the eye with occasional spread to the lacrimal duct. Ptosis may be a presenting complaint [14]. If the initial bite occurs on the conjunctival mucosa, the disease is termed mucocutaneous leishmaniasis. This state may lead to severe ulceration and possible loss of the eye. 

The diagnosis of leishmaniasis is made by direct demonstration of organisms on tissue smears or biopsy. Amastigotes are usually demonstrated fairly easily in the case of cutaneous or mucocutaneous ocular disease. However, amastigotes have not been directly identified in cases of ocular disease associated with kala-azar. When present, *Leishmania* spp. may be cultured on Novy, MacNeal, Nicolle's medium (N.N.N.) as well as Schneider's *Drosophila* medium supplemented with 30% fetal bovine serum. While available, serologic testing is not particularly useful for diagnosing cutaneous and mucocutaneous disease due to cross-reactivity with *T.cruzi* and *Mycobacterium leprosum*.

Treatment of choice is pentavalent antimony, sodium stibogluconate 15–20 mg per kg per day IM or IV for 15–20 days. A second or even a third treatment course with pentavalent antimonal can be given over 6–8 weeks if healing is not progressive. 

### 2.5. Malaria

Caused by the *Plasmodium* species and transmitted via the bite of the female anopheles mosquito, this sometimes fatal infectious disease has characteristic findings in the eye. Signs of *falciparum* malaria in the eye include retinal whitening, retinal haemorrhage, papilloedema, and cotton wool spots [15, 16]. Much research done in endemic areas has shown a correlation between papilloedema or extramacular retinal oedema (retinopathy) and poor outcome in children with cerebral malaria. Studies show that retinopathy was associated with subsequent death, and the increasing severity of retinal signs was related to increasing risk of fatal outcome [17]. Other studies have shown that retinal changes related to microvascular obstruction were common in adults with severe falciparum malaria and correlated with disease, severity and comma [18, 19]. It is important to emphasise that whilst these signs give a pointer to the severity of disease they do not alter the drug management of malaria. The outcome in terms of vision in patients with ophthalmological findings and severe malaria is usually good [20]. Insights from retinal investigations have furthered the understanding of cerebral malaria [21, 22]. 

Quinacrine and chloroquine are molecules with the same alkyl side chain but different nuclei. The photobiological effects of quinacrine and chloroquine are similar in model systems; thus, development of a bull's-eye maculopathy with quinacrine ingestion is an unsurprising potential side effect. 

The definitive diagnosis of malaria is made by microscopic identification of the parasite in the blood smear. A thin blood film should be examined for at least 15 minutes, whereas a 5-minute search of a thick film should reveal parasites if present. The thick film is the most efficient method of detecting malarial parasites, but interpretation requires an experienced worker. 

Antimalarial drugs may be classified as (1) suppressive, by acting upon asexual blood cell stages and preventing the development of clinical symptoms; (2) therapeutic, by also acting on asexual forms to treat the acute attack; (3) radical cure, for destruction of the EE forms; (4) gametocytocidal, for destroying gametes; (5) sporoniticidal, for drugs that render gametocytes noninfective in the mosquito. 

### 2.6. Microsporidiosis

Two genera appear to be important in the pathogenesis of ocular disease: *Encephalitozoon* and *Nosema*. Another genus, *Microsporidium*, is classified as *Nosema*-like. Recently, *Septata* spp. has been implicated in keratoconjunctivitis [23]. It should be noted that knowledge of microsporidiosis seems to be rapidly expanding, given its important role as an opportunistic infection in patients with AIDS. The life cycle is somewhat complex, involving three general stages: infection, merogony, and sporogony. 

Ocular infection is presumed to occur either by direct inoculation into eye structures or by dissemination systemically, with the latter proposed to be the pathogenesis in patients with AIDS. Ocular findings are generally limited to the conjunctiva and cornea. 

With respect to diagnosis, spores have been demonstrated in most cases in which corneal scrapings or biopsy specimens are examined by light or electron microscopy. Where available, serologic testing may assist in the diagnosis of microsporidiosis. 

Current recommendations for treatment include the use of albendazole, which has shown some promise in the treatment of corneal disease. Historically, severe, progressive cases of ocular microsporidiosis have resulted in enucleation.

### 2.7. Rhinosporidiosis

Rhinosporidiosis, caused by *Rhinosporidium seeberi*, is a mucocutaneous disease that involves the palpebral conjunctiva in ~15% of all cases of rhinosporidiosis [24]. It is an infrequent cause of disease in India and tropical South America. Reproduction of *R.seeberi* in tissue produces polypoid or papillary growths that arise from mucous epithelium. Recent investigations of RNA genes from this microorganism disclose that it may be more closely related to fish parasites than to fungi [25], and it is, therefore, included in protozoan diseases of the eye. The etiologic agent, *Rhinosporidium seeberi,* has never been successfully propagated in vitro. 

At present, the treatment for rhinosporidiosis is the surgical excision. Some authors proposed a medical therapy with dapsone [26], but the results are not convincing. Antimicrobial therapy is ineffective, and the disease may recur after months or years. 

### 2.8. Toxoplasmosis

Toxoplasma gondii is a protozoan parasite, the lifecycle of which passes through cats. It represents the commonest cause of uveitis worldwide [27]. Human infection occurs through ingestion of food or water contaminated with cat faeces. Toxoplasmosis may be acquired at any age but most commonly during childhood. The majority of infections are asymptomatic and the prevalence of antitoxoplasma IgG (indicating past infection) ranges from 15 and 20% in Northern Europe to 80% or more in parts of the developing world. South America has a particularly high rate of ocular disease from toxoplasmosis.

Some patients may present with a glandular fever-like systemic febrile illness with adenopathy. Most cases of adult infection will not present with eye signs, those that do usually present with a focal necrotising retinitis occasionally associated with vascular occlusion [28]. Toxoplasmosis in immunosuppressed patients, for example, with AIDS can present with multiple, widespread lesions of differing chronicity and look different from classical toxoplasmosis. Vitritis is a common feature of toxoplasmosis which often leads to symptomatic haze and floaters that lasts for months after the resolution of the acute attack. Lesions are usually self-limiting, but where they threaten sight—around the macula or optic nerve—treatment with a combination of corticosteroids, pyrimethamine, and sulfadiazine is usually advocated. However, therapeutic trials suggest that there is little evidence that drug therapy alters the natural history of the disease [29]. Relapses are relatively common, occurring in around 80% of patients followed up for more than 5 years [30]. The most serious consequences of toxoplasmosis are seen when acquisition occurs in pregnancy leading to congenital infection of the newborn. Involvement of the macula is common in the developing foetus and has devastating effects on central vision. Bilateral ocular involvement is common, and both maculae can be affected. 

Serologic tests are very important in the diagnosis of toxoplasmosis. Because of the common occurrence of antibodies to the parasite in the general population, diagnosis by serologic means requires the demonstration of a significant increase in antibody titers. 

Drug treatment for ocular and cerebral toxoplasmosis is the same and lesions will continue to grow without therapy. Clindamycin and azithromycin are now commonly used as first line treatment. Azithromycin has been shown to be effective in reducing the number of attacks in Brazil (see Tables 1 and 2).

## 3. Helminthic Eye Infection

### 3.1. Eye Infection Caused by Round Worms

#### 3.1.1. Angiostrongyliasis

Ocular angiostrongyliasis, caused by *Angiostrongylus cantonensis*, is a nonfatal disease; however, it can cause permanent damage to an affected eye. It was first reported in Thailand in 1962 [31] and since then has rarely presented in tropical countries [32]. According to the *A. cantonensis* life cycle, the human is an accidental host. Most larvae usually live in the subarachnoid space or brain parenchyma. Only a small number of worms remigrate to pulmonary arteries or move randomly to other tissues such as cranial nerves or orbits.

Although blood eosinophilia is demonstrated in most cases of eosinophilic meningitis [33], it has not been noticed in ocular angiostrongyliasis without eosinophilic meningitis. Hence, it may indicate that ocular angiostrongyliasis occurs because a worm moves randomly from the bloodstream to an eye without invasion of the brain or meninges. In cases of ocular involvement ocular symptoms usually present between 2 weeks and 2 months after snail ingestion. 

Although a wide range of initial visual acuity was reported, from finger count to 6/6, five cases had visual acuity less than 2/60. The duration of visual impairment varied from 4 days to 8 weeks, mostly 2-3 weeks. Additionally, indirect ophthalmoscopy should be recommended in any individual presenting with a history of eating raw *Pila* spp. snails and blurred vision either with or without eosinophilic meningitis. No dominant affected eye has been reported because of a nonspecific pattern of parasite movement [34].

Any types of laser, surgical removal, and corticosteroid treatment did not improve visual acuity. Alteration of the retinal pigment epithelium or retinal inflammation caused directly by parasites was the main reasons for poor vision at presentation. Furthermore, it produced permanent damage to an affected eye and gave a poor outcome. Although corticosteroids and albendazole have been reported to be effective in ocular cysticercosis and neurocysticercosis [35], there are no specific anthelmintic therapies in ocular angiostrongyliasis even after a definite diagnosis has been made.

#### 3.1.2. Bancroftian and Brugian Filariasis

Human ocular infestation by live filarial worm is a rare occurrence and has been reported mostly from South-East Asia. It involves the eyelids, conjunctiva, cornea, anterior chamber, and uvea. Ocular filariasis can present in an otherwise asymptomatic patient without any constitutional symptoms. Inflammation of the retinal pigment epithelium and retinal vasculitis decreased vision, and panuveitis with secondary glaucoma can occur. 

Indirect ophthalmoscopy showed vitritis with plenty of vitreous membranes, and subretinal yellow lesions in the peripheral retina along with retinal pigment epithelial tracts [36]. An aqueous tap and a peripheral blood smear isolate microfilariae of *W. bancrofti*. Therapy with diethyl carbamazine citrate along with systemic steroids provides symptomatic relief. 

#### 3.1.3. Baylisascariasis

*Baylisascaris procyonis* is the common intestinal raccoon roundworm in North America and is found in 82% of raccoons in Illinois. It is a known cause of neural larva migrans in animals [37]. It was identified in seven childhood cases manifesting as diffuse unilateral subacute neuroretinitis and choroidal infiltrates in association with neurologic disease. Those children had a history of pica and raccoon exposure. Differences in inoculum level are likely responsible for isolated ocular larva migrans versus neural larva migrans in humans [38]. 

Identification of the worm in the eye is the definitive diagnosis. Indirect immunofluorescence assays on serum, and cerebrospinal fluid is usually positive or serially positive and increasing [39]. Treatment is with albendazole and corticosteroids, and prognosis is usually poor. 

#### 3.1.4. Dirofilariasis

Dirofilaria are parasitic nematodes that are common in domestic and wild animals. Dirofilarial zoonotic infections are caused by mosquito vectors that carry the parasites from their animal hosts to people. Although these infections remain rare, they are increasing in incidence and human dirofilariasis may be considered an “emergent zoonosis [40]. 

As the worm matures, it elicits a host inflammatory response that ultimately produces the clinical presentation of a subcutaneous nodule. These nodules are most often found on areas with exposed skin [41]. Subcutaneous dirofilariasis appears as a small subcutaneous nodule that gradually grows over periods of weeks or months. The consistency of the nodule is hard and elastic with marked erythema. When the location is ocular, the worms are situated in the conjunctiva and can be extracted by incision.

The diagnosis of dirofilariasis is established histopathologically. Both the gross and microscopic features of *D. tenuis* have been well described [42]. Once diagnosed, the recommended treatment is complete removal of the nematode. If the nematode is not removed, it eventually degenerates, and the mature granulomatous response results in either calcification or abscess formation with subsequent purulent expulsion of the parasite. 

#### 3.1.5. Loiasis

The agent of loiasis is *Loa loa*. Infection is acquired by humans through the bite of the tabinid flies of the genus *Chrysops*. When humans are bitten, larvae pass from the fly to the human, where they develop over 1year into mature adult worms [43]. These adults migrate through cutaneous and deep connective tissue, producing microfilariae. Ocular disease may be due to both the presence of microfilaria and the presence of the adult worm. 

The diagnosis of loiasis is generally made by the detection of circulating microfilariae. In cases of conjunctival involvement, extraction of an adult worm confirms the diagnosis. Therapy of loiasis involves the manual removal of adult worms present in the conjunctiva in addition to the use of diethylcarbamazine (DEC). Severe hypersensitivity responses may occur due to the killing of both microfilariae and adult worms.

#### 3.1.6. Onchocerciasis (River Blindness)

It appears that humans are the main reservoir of onchocerciasis, with infection occurring from the bite of an infected female blackfly, *Simulium* spp. that require fast-flowing water for their breeding and development. The disease is restricted to areas adjacent to river systems. An estimated 37 million people in 34 countries in Sub-Sahara Africa and South America are affected by it [44]. After biting an infected person and ingesting microfilariae, the microfilariae mature to the larval stage as they migrate to the proboscis of the fly. There, the larvae may be injected into a human with the next bite, resulting in the formation of an adult worm capable of producing microfilariae. These microfilariae migrate throughout skin and connective tissue, where they die after several years. Adult worms may live in the subcutaneous tissue for years, with a female producing one-half to one million microfilariae yearly. The site of the adult worm is usually found over a bony prominence and may develop into a firm, nontender nodule, or onchocercoma. 

It is the migration of microfilariae through skin and connective tissue which is responsible for the majority of clinical findings in onchocerciasis. Ocular onchocerciasis is due to the presence and/or migration of microfilariae in and through ocular structures as well as the host's response to the migration [45]. There are five predominant ocular findings that correlate with the location of microfilariae: punctate keratitis, sclerosing keratitis, iridocyclitis, chorioretinitis, and optic atrophy. Other findings may include distortion of the pupil, which may also be covered with exudate. 

Wolbachia and Wolbachia-derived molecules are bacterial symbionts of *O. volvulus* that is implicated in the pathogenesis. Experiments using Wolbachia-containing extracts of *O. volvolus* in a mouse model of onchocercal keratitis demonstrated that the presence of the bacteria was essential for neutrophil-mediated inflammation, opacity, and corneal haze [46]. 

The diagnosis of onchocerciasis is accomplished by a combination of clinical symptoms and signs with histopathologic examination of specimens. Slit lamp examination may confirm the presence of microfilariae in the anterior chamber. A sclerocorneal punch biopsy may aid in the diagnosis as well [47]. Rarely, microfilariae are demonstrated in blood and/or urine samples. PCR may aid in the diagnosis of disease associated with a low burden of microfilariae. Xenodiagnosis, using laboratory-bred blackflies, may provide a clue as well. 

Traditional therapy has centered on the use of DEC, but this is active only against microfilariae, allowing adult worms to repopulate the microfilariae in several months. 

Ivermectin is the treatment of choice and mass distributed by the WHO Onchocerciasis Control and the Onchocerciasis Elimination Programme for the Americas. This had led to dramatic improvements in disease control to the extent that elimination has become a realistic target [48].

#### 3.1.7. Thelaziasis

Transmission of eyeworms occurs via nonbiting diptera that feed on the ocular secretions, tears, and conjunctiva of animals. The disease, thelaziasis, is characterized by a range of subclinical to clinical signs such as epiphora, conjunctivitis, keratitis, corneal opacity, and ulcers [49]. The adult and larval stages are responsible for eye disease. Asymptomatic, subclinical thelaziasis occurs mainly when only the male nematodes parasitize animals, whereas evident symptoms have been more frequently registered in the presence of gravid females [50]. The lateral serration of the *Thelazia* cuticle causes mechanical damage to the conjunctival and corneal epithelium. 

Collected nematodes are identified based on morphologic key [51]. *T. callipaeda* nematodes have a serrated cuticle, buccal capsule, mouth opening with a hexagonal profile, and 6 festoons. Fortreatment of human cases, the removal of the worm is suggested. Topical treatment with thiabendazole hasalso been reported to kill the worms.

#### 3.1.8. Toxocariasis

Larva migrans in man are a disease characterized by inflammatory reaction around or in the wake of migrating larvae, most commonly larvae of nematode parasites of other animals. For some of the larva migrans producing larvae, man is merely an accidental but more or less normal intermediate or paratenic host. 

Toxocariasis is an important cause of unilateral visual loss and leukocoria in infants, and as a differential diagnosis of retinoblastoma. *Visceral larva migrans* are best known in the form produced by the larvae of *Toxocara canis*, these having been identified in autopsy specimens of lungs, liver, brain, and in several enucleated eyes [52]. Human infection by a spiruroid form of nematode *Gnathostoma spinigerum* has been reported sporadically from Thailand, the Philippines, China, Japan, and India. The high prevalence may be increasing in areas whereby freshwater raw fish is customary. Palpebral oedema with conjuctival erythema developed when lesions developed near the eye. Intraocular parasites occur so rarely that they are considered as ophthalmological curiosities, nevertheless, it can cause intraocular hemorrhage, uveitis, and loss of vision within 2 days [53]. Following surgical removal, treatment is with albendazole and topical corticosteroids. 

#### 3.1.9. Trichinosis

Trichinosis is a parasitic disease which probably presents itself for diagnosis not infrequently. Because of its varied symptomatology trichinosis is, unless by chance, almost as frequently undiagnosed. This is evidenced by the comparatively few cases reported in the literature. Ocular trichinosis can manifest itself as oedema of the face especially around the eyes, conjunctivitis, and exophthalmoses. Diagnosis is only confirmed by finding the worm in a section of the excised muscle (see Tables 3 and 4). 

### 3.2. Eye Infection Caused by Flat Worms

#### 3.2.1. Cysticercosis

This helminthic infection caused by the larval cysts of the pork tapeworm (*Taenia solium*). Infection is often asymptomatic though neurological symptoms—predominantly seizures—are the most common manifestation. Ocular involvement is well recognised and includes orbital, intraocular, subretinal, and optic nerve lesions [54–57]. Cysticercosis can be evident as a free-floating cyst with amoeboid movements within the vitreous or anterior chamber of the eye. Gaze palsies may also occur secondary to intramuscular cysts or cranial nerve lesions from intracerebral cysts. 

Diagnosis depends on imaging with ultrasound, MRI, and CT scanning all being useful, depending on the location of the cysts [58, 59]. Serology can be useful but in cases of isolated cysts may be negative. 

Treatment is largely with the antihelminthic albendazole. Antihelminthic therapy may lead to an increased inflammatory reaction around the lesions, and for this reason corticosteroids are often used when treating neurological or ocular disease. Spontaneous extrusion of cysts from the orbit may occur, and surgery may be required for isolated ocular lesions when they are growing and causing visual loss. 

#### 3.2.2. Fascioliasis

*Fasciola hepatica* is a zoonotic helminth that is prevalent in most sheep-raising countries. In 1989, an outbreak of human infestation of more than 10 000 cases living in Guilan Province, Iran was reported [60]. The biliary duct of the liver is the main site of establishment of the parasite. However, immature flukes may deviate during migration, entering other organs, and causing an ectopic infestation [61]. In humans, ectopic locations in the orbit [62, 63] have been reported. Identification of the route of entry of the parasite larva into the anterior chamber of the eye is difficult. One possible route can be via the central retinal artery into the vitreous, causing vasculitis and endophthalmitis [64]. Severe intraocular reaction, haemorrhage, diffuse vasculitis, and retinal ischaemia of the patient may be caused as a result of the presence or irritation of the parasite. Early vitrectomy and removal of the parasite resulted in a rapid response, with reasonable final visual acuity. 

#### 3.2.3. Hydatid Cyst

Hydatid cysts are most commonly seen in the liver (60–70%) and lungs (20%) [65]. Hydatid disease involving the orbit represents <1% of all cases of hydatid disease [66] and requires surgical treatment. 

Definitive preoperative diagnosis is difficult [67]. Laboratory and immunologic tests are generally unhelpful. From the literature and our own observations, orbital hydatid cysts usually appear as a well-defined, thin-walled, oval shape lesions with fine peripheral rim enhancement of their fibrous capsule after contrast medium administration [68]. 

#### 3.2.4. Schistosomiasis

Various theories have been postulated as to the different routs by which the schistosoma ova or even the adult worms can reach the systemic circulation and then after lodged in ectopic sites such as the eyes. Cercariae (the infective stage) develop to maturity and lay their eggs in the veins directly under the skin or the mucous membrane through which they have penetrated if the part is richly vascularised [69]. The presence of schistosomal eggs in the eye can produce granuloma formation and inflammatory sequelae [70]. Considering how common the infection is in endemic areas, involvement of the eye is incredibly rare. 

Effective treatment, using the drug praziquantel, has been available for 25 years, but the growth of human populations in high-risk areas, as well as the high probability of rapid re-infection after treatment, has thwarted efforts to control the number of human infections worldwide [71] (see Tables 5 and 6).

## 4. Ocular Infestation Caused by Ectoparasites

### 4.1. Myiasis

Ocular myiasis is the result of invasion of the eye by larvae of flies. Genera important to human myiasis include *Dermatobia*, *Gasterophilus*, *Oestra*, *Cordylobia*, *Chrysomia*, *Wohlfahrtia*, *Cochliomyia*, and *Hypoderma*. Ophthalmomyiasis may be categorized into three categories: ophthalmomyiasis externa, ophthalmomyiasis interna, and orbital myiasis [72]. 

Ophthalmomyiasis externa is usually seen in areas of shepherding and is typically due to larvae of the sheep nasal botfly, *Oestra ovis* [73]. A crawling or wriggling sensation accompanied by swelling and cellulitis may be seen in palpebral myiasis. 

Ophthalmomyiasis interna is most commonly caused by a single larva of the *Hypoderma* spp. Infection is due to invasion of the tissues, leading to uveitis. More serious complications may include lens dislocation and retinal detachment [74]. 

Orbital myiasis may be due to a number of fly species and is generally seen in patients who are unable to care for themselves [75]. 

Diagnosis of ophthalmomyiasis is made by demonstration of maggots, and histologic examination may show granuloma formation. Anticholinesterase ointment may help kill or paralyze the larvae. Steroids and antibiotics may be necessary to control inflammation and secondary bacterial infection.

### 4.2. Phthiriasis Palpebrum

Lice belong to the order Anoplura. Of these, medically important species include *Pediculus humanus* var. *corporis*, the human body louse; *Pediculus humanus* var. *capitis*, the human head louse; *Phthirus pubis*, the crab louse. Depending on the species, eggs, or nits, are laid and glued to body hairs or clothing fibers. Following this, nymphs emerge to feed on the host, giving rise to symptoms of pruritis. Of the species mentioned, *P.pubis* is most likely to involve the eyebrows and eye lashes. In addition to pruritis, small erythematous papules with evidence of excoriation may be present. Involvement of the eyelash may cause crusting of the lid margins. In this case, diagnosis is relatively simple as nits are easily seen at the base of the eyelash [76]. 

Eyelid disease is treated with a thick layer of petrolatum twice a day for 8days, or the application of 1% yellow oxide of mercury four times a day for 2  weeks [77]. 

### 4.3. Tick Infestation

Ticks are arthropods belonging to the class Arachnida. There are a number of different species of ticks which may cause disease in humans and animals. Ticks exist in three life stages—larva, nymph, and adult—all of which requires blood meals. Most tick bites are uncomplicated, and prompt removal of the tick is all that is necessary [78]. Ticks have been reported to attach to ocular structures. In one such case, the nymph was associated with a stinging sensation [79]. Following the removal of the tick, a firm nodule, representing a tick bite granuloma, may remain for several weeks. This granuloma likely represents retained tick material and generally resolves spontaneously. 

## 5. Search Strategy and Selection Criteria

This is a comprehensive paper of the parasitic diseases/infections of the eye. The majority of the clinically important species of parasites involved in eye infections are reviewed in this paper. Parasites are discussed by the disease or infection they cause. Emphases have been placed on literatures published within the past decade, but prior noteworthy reviews and case reports are included. 

We searched the MEDLINE database via PubMed and identified articles by cross-referencing the terms ocular, eye, ophthalmic, retinitis, endophthalmitis, conjunctivitis, and uveitis to specific infectious diseases in adults. We searched the Cochrane database for systematic reviews on the treatment of specific parasitic ocular infections. Additionally, we reviewed texts for completeness and to obtain other references of eye complications of systemic infections (see Tables 7 and 8). 

## Figures and Tables

**Table 1:** Ocular parasitosis caused by protozoa (*geographical distribution & ocular findings*).

Disease/Infection	Causative agent	Geographical distribution	Ocular findings
Acanthamoebic keratitis	*Acanthamoeba* spp	Worldwide, soil and water	Conjuctival oedema, sever pain, ring infiltrate around the cornea, hypopyon, hyphema, uveitis, loss of vision

Chagas' disease	*Trypanosoma cruzi*	Central and South America	Palpebral and periorbital oedema

Giardiasis	*Giardia lamblia*	Southeast Asia, Europe, USA and South Africa	Salt and pepper retinal changes, chorioretinitis, retinal haemorrhage and uveitis

Leishmaniasis	*Leishmania* spp	Africa, Mediterranean region, Middle East, parts of Asia and Central and South America	*Visceral*: conjunctivitis, uveitis and retinal haemorrhage *Cutaneous*: lesions on eyelid, blepharoconjunctivitis *Mucocutaneous*: severe ulceration, loss of the eye

Malaria	*P*. *falciparum *	Africa, Central and South America, Oceania and Asia	Retinal haemorrhage, papilloedema, cotton wool spots

Microsporidiosis	*Microsporidia* spp	Worldwide	Conjunctival hyperemia, punctate epithelial keratitis, hyphema, necrotizing keratitis, corneal ulcer

Rhinosporidiosis	*R*. *seeberi *	South America and Africa	Cojuctival granuloma

Toxoplasmosis	*Toxoplasma gondii*	Worldwide, South America	Congenital: strabismus, nystagmus and blindness Acute aquired: Primarily; necrotizing chorioretinitis Virtitis is common Secondary findings include; scotoma, photophobia, blindness, Glaucoma, ↑ IOP, necrotizing inflammation, loss of central vision

**Table 2:** Ocular parasitosis caused by protozoa (*diagnosis and treatment*).

Disease/Infection	Diagnosis	Treatment
Acanthamoebic keratitis	Corneal scrapings, culture	Propamadine (0.1% solution) + antibacterial preparation, polyhexamethylene biguanide (0.02% solution), chlorhexidine (0.02% solution), keratoplasty.

Chagas' disease	Blood smear, Buffy coat*, culture, Xenodiagnosis*	Nifurtimox, benznidazole

Giardiasis	Diagnosing intestinal disease and exclusion	Metronidazole, albendazole, paromomycin

Leishmaniasis	Tissue smears or biopsy, culture in NNN medium	Antimonials, amphotericin B, Paromomycin, Fluconazole, zinc sulfate

Malaria	BFMP*, Buffy coat, PCR, serological	Chloroquine, Primaquine, Dapsone, Mefloquine, artemisinin derevatives

Microsporidiosis	Corneal scrapings, biopsy, serological	Albendazole

Rhinosporidiosis	Histopathologic demonstration	Dapsone, amphotericin B

Toxoplasmosis	Serology (IgM, IgG), PCR	Clindamycin+ azithromycin, pyrimethamine+ sulfadiazine,

*Buffy coat: The thin layer of concentrated white blood cells that forms when a tube of blood is spun in a centrifuge.

*Xenodiagnosis: a method of animal inoculation using laboratory-bred bugs and animals.

*BFMP: Blood Film for Malaria Parasite.

**Table 3:** Ocular parasitosis caused by round worms (*geographical distribution & ocular findings*).

Disease/Infection	Causative agent	Geographical distribution	Ocular findings
Angiostrongyliasis	*Angiostrongylus cantonensis *	Southeast Asia, Pacific region, eastern Australia	Blurred vision and poor visual acuity

Bancroftian and Brugian filariasis	*W. bancrofti, B. malayi*	Southeast Asia	Retinal vasculities, decreased vision and panuveitis with secondary glaucoma

Baylisascariasis	*Baylisascaris procyonis *	Few records in US, Japan, Germany	Vision loss, transient visual obscuration, and diffuse unilateral subacute neuroretinitis

Dirofilariasis	*Dirofilaria repens *	Europe, Asia and Africa	Pain, oedema, and congestion of the conjunctiva, diplopia, foreign body sensation in the eye

Loiasis	*Loa loa *	Central and West Africa	Conjunctival congestion and pain with movement of the eye. May affect vision transiently. Retinal hemorrhages and perivascular inflammation

Onchocerciasis	*Onchocerca volvulus *	Tropical Africa, South America, and the Arabian peninsula	Chorioretinitis, keratitis, uveitis, corneal opacification, neovascularisation, blindness

Thelaziasis	*Thelazia callipaeda *	Asian Pacific region	Epiphora, conjunctivitis, keratitis, corneal opacity and ulcers

Toxocariasis	*Toxocara canis *and* Toxocara cati *	Wide spread	Peripheral white mass is often visible in affected eyes

Trichinosis	*Trichinella spiralis *	Central and eastern Europe, united States	Oedema around the eye, conjunctivitis and exophthalmoses

**Table 4:** Ocular parasitosis caused by round worms (*diagnosis and treatment*).

Disease/Infection	Diagnosis	Treatment
Angiostrongyliasis	Identification of *Angiostrongylus cantonensis* in the eye Direct and indirect immunofluorescent	Oral and topical prednisolone, laser treatment, surgical removal of the parasite

Bancroftian and Brugian filariasis	An aqueous tap and a peripheral blood smear isolate microfilariae or adult worm	Carbamazine citrate along with systemic steroids

Baylisascariasis	Exclusion of other known causes of OLM*	Steroids and antihelminthic agents

Dirofilariasis	Excision biopsy	Surgical excision of the adult worm, DEC*

Loiasis	Extraction of adult worm or microfilaria	Manual removal of adult worm or microfilaria present in the conjunctiva and DEC

Onchocerciasis	Slit lamp, sclerocorneal punch biopsy, Xenodiagnosis	Manual removal of adult worms, ivermectin or mebendazole

Thelaziasis	Eggs or larvae can be seen when tears or other eye secretions are examined under light microscope	Surgical

Toxocariasis	Serology	Cryopexy and photocoagulation, albendazole and corticosteroid

Trichinosis	Muscle biopsy	Thiabendazole, mebandazole, steroids

*OLM: ocular larva migrans.

*DEC: diethylcarbamazine.

**Table 5:** Ocular parasitosis caused by flat worms (*geographical distribution & ocular findings*).

Disease/Infection	Causative agent	Geographical distribution	Ocular findings
Cysticercosis	*Cysticercus cellulosae *	Mexico, Central America, Indian subcontinent, Far East and Africa	Subconjunctival and eyelid masses, papilloedema, cranial nerve palsies, vitritis and optic neuritis

Fascioliasis	*Fasciola hepatica *	Africa and Asia	Painful red eye, and there may be visual defect

Hydatid cyst	*Echinococcus granulosus *	South America, Australia, Middle East and Mediterranean countries	Orbital swelling, exophthalmus and proptosis

Schistosomiasis	*S. mansoni,* * S. haematobium,* * S. japonicum*	Sub-Sahara Africa, China, South Asia	Uveitis and subretinal granuloma

**Table 6:** Ocular parasitosis caused by flat worms (*diagnosis and treatment*).

Disease/Infection	Diagnosis	Treatment
Cysticercosis	Imaging with ultrasound, MRI and CT Serology can be useful	Albendazole, corticosteroids
Fascioliasis	Adult worm in the eye	Vitrectomy and removal of the parasite
Hydatid cyst	Imaging	Surgical removal
Schistosomiasis	Eggs in the feces, urine or eggs/cercariae in the eye	Praziquantel

**Table 7:** Ocular parasitosis caused by ectoparasites (*geographical distribution & ocular findings*).

Disease/Infection	Causative agent	Geographical distribution	Ocular findings
Myiasis	Larvae of flies	Shepherding places	Uveitis, lens dislocation and retinal detachment
Phthiriasis palpebrum	*Phthirus pubis *	Cosmopolitan	Crusting of the eyelid margins
Tick infestation	Hard and soft ticks	Cosmopolitan	Stinging sensation

**Table 8:** Ocular parasitosis caused by ectoparasites (*diagnosis and treatment*).

Disease/Infection	Diagnosis	Treatment
Myiasis	Demonstration of maggots, histological examination	Manual removal of the maggots, anticholinesterase ointment
Phthiriasis palpebrum	Nits seen at the base of eyelashes	Manual removal of the lice and nits, thick layer of petrolatum, 1% yellow oxide of mercury
Tick infestation	Biomicroscopy may reveals ticks embedded in conjunctiva, eyelid margins or crawling on the eyelashes	Removal by conjunctival excision
